# English validation of the Multidimensional Scale of Motives for Postponing Parenthood (MSMPP-18-EN): Factorial structure, psychometric properties, and correlates

**DOI:** 10.1371/journal.pone.0329404

**Published:** 2025-08-18

**Authors:** Małgorzata Szcześniak, Adam Falewicz, Marianna Chmiel, Zdzisław Kroplewski

**Affiliations:** Institute of Psychology, University of Szczecin, Szczecin, Poland; Zurich University of Applied Sciences: ZHAW Zurcher Hochschule fur Angewandte Wissenschaften, SWITZERLAND

## Abstract

**Introduction:**

While the literature on deferred parenthood is rich in analyses of this topic from a sociological and medical point of view, psychological research is in the minority. The analysis also shows that there are no questionnaires to measure motives for postponing parenthood. This gap is filled by the Multidimensional Scale of Motives for Postponing Parenthood (MSMPP-18) which assesses the motivational forces that may lead to the decision to postpone parenthood. Given that most studies and articles on deferred parenthood are reported in English, the two main goals of Studies 1–3 reported in the present research were to: 1) validate the original Polish version of the MSMPP-18 into English; 2) confirm its convergent validity.

**Methods:**

The original version of the MSMPP-18 was translated into English by two independent psychologists fluent in academic English using a traditional forward-backward translation technique. The factorial structure of the MSMPP-18-EN and its psychometric characteristics were verified through Confirmatory Factor Analysis (CFA). The criterion validity of the scale was examined using the correlation between the motives for postponing parenthood and a nomological set of variables in Studies 1–3 (total *N* = 664; *n*_*1*_ = 247; *n*_*2*_ = 239; *n*_*3*_ = 178).

**Results:**

The CFA statistics provided empirical evidence that the MSMPP-18-EN has good fit indices across Studies 1–3, both for the first-order model and the second-order model. The research confirmed that the English-language version of the scale reveals factors analogous to the original scale: 1) feeling of uncertainty and incompetence; 2) self-focus; 3) parenthood as a burden; 4) fear of change; 5) financial security concerns; and 6) worry about a child’s future. The values of Cronbach’s alpha (Studies 1–3: 0.75–0.95; 0.68–0.93; 0.77–0.93), McDonald’s Omega (Studies 1–3: 0.76–0.96; 0.73–0.93; 0.79–0.93), and CR (Studies 1–3: 0.89–0.97; 0.80–0.99; 0.78–0.99) displayed good internal reliability. Data from Studies 1–3 also showed that procrastination, future anxiety, need for closure, negative emotions toward God, and family disfunction positively and significantly correlated with motives for delayed parenthood and its overall score. On the other hand, motives of postponed parenthood were negatively and significantly correlated with psychological capital, social support, positive emotions toward God, life satisfaction, self-efficacy, and self-regulation.

**Conclusions:**

The presented validation of what is probably the first scale measuring the motives for deferred parenthood allows us to assume that the MSMPP-18-EN tool in the English version meets the theoretical and empirical criteria of a good questionnaire.

## Introduction

Postponed parenthood, also referred to as delayed childbearing or deferred parenthood, is a phenomenon that consists in “the increase in the average age of women having their first child” [1, p. 651]. Although there is a lack of consensus as to the definition of postponed parenthood [2], in the scientific literature, delayed childbearing alludes to a first birth to a woman aged 30 years [3], 35 years, or even 40 or over [2].

Recent shifts in marriage and the late transition to parenthood have become a global social issue [4] as it affects many countries around the world, bringing social, health, demographic, and economic consequences. For example, the trend toward deferred parenthood is rising in the United States, where the National Vital Statistics Report for 2022 shows that “birth rates fell 7% for women ages 20–24, rose 1% to 5% for women ages 25–29 and 35–44, and rose 12% for women ages 45–49” [5, p. 1]. It is also on the rise in Canada [6], where women are tending toward later marriage and fewer children [7]. Childbearing delay is a widespread characteristic among Australians [8] and other English-speaking countries such as the United Kingdom [9], Ireland [10], New Zealand [11], and Nigeria with the largest population of women in the African continent [12]. The phenomenon of postponing parenthood until later years has also been observed in the high-income and low-fertility societies in Europe and East Asia [13,14]. An estimation by Statista [15] shows that the highest average age of women upon the birth of their first child in Europe was found in Spain (31.6 years old) and Italy (31.7 years old). As reported by Chen [13], the highest median age of first-time mothers in the world was noticed in Hong Kong (32.7 years old) and South Korea (32.8 years old).

As the literature shows, postponing parenthood has various causes and is often related to the prioritization of alternative life goals [16]. Zabak et al. [17] connect delayed parenthood with the changing aspirations, expectations, and opportunities of women and men today. Previous research indicates that women entering parenthood at a later time is associated with educational attainment [18], career path [19] and occupational status [20].

Changes in social norms are another pertinent factor of postponed parenthood. Sobotka [21] notes that we are currently witnessing rapid changes in family norms and values. Unlike the goals that young people set for themselves 50 years ago, nowadays, family is not the primary objective of their contemporary peers. Moreover, the stories of adult Swedes interviewed by Bodin et al. [22] confirm that, although people still want to start new families, they make great efforts to have everything they need before having children and “to be as prepared as possible for adverse events” [p. 14].

A delayed decision to become a parent may also be related to the family background. According to Nisén et al. [23], Finnish women coming from urban families with high socioeconomic status and fewer siblings tend to have children later than their less affluent counterparts. In another study [24], Waldenström points out that a less-than-positive experience of one’s own mother and father is a predictor of being childless at age 32 among Swedish and Norwegian respondents. Pöyliö and Van Winkle [25] specify that high-income women from underprivileged backgrounds defer entry into parenthood in Finland and the United States.

Voluntary adoption of contraception and involuntary difficulties in getting pregnant are a separate category of factors related to the later decision to become a parent. Several studies sustain that different birth control methods affect family planning worldwide [26,27]. Moreover, health problems (e.g., autoimmune diseases or metabolic problems) are mentioned as one of the main reasons for delay in childbearing [28,29]. This is due to the fact that the diagnosed disease requires complex and often long-term treatment to enable pregnancy.

The lack of a suitable partner [30,31], as well as partnership instability and disagreement [32] are widely considered to be important reasons for delaying the birth of a first child. Some young people acknowledge that they do not have a committed partner who shares their desire for parenthood or that they are not ready to “jump into having kids” themselves [33, p. 867]. Meeting the right partner [33] who has similar beliefs about family, who can be trusted and who is “at the same phase of life” [26, p. 331] is often mentioned by respondents as a factor accelerating the decision to have a child.

Material factors are the last but not least group of reasons that contribute to later parenthood. Various authors [10,27] note that economic uncertainty, employment instability, low availability and high housing prices prevent young people from making early decisions about having their first child. As Beaujouan and Sobotka [27] observe, worse economic conditions often result from weak social policies.

As results from the above-mentioned examples, the causes of delayed family formation are related to a constellation of several different factors: personal choice, expansion of university education, career, social norms, family background, rise of effective contraception, illness, forming a lasting partnership, economic uncertainty, precarious labor markets, housing conditions, and social polices [4,10,24,34–37].

Although the reasons presented above show a complex pattern of motives for why people postpone starting a family, they do not exhaust the whole range of factors. There are a number of motives that connect with anthropological vision, religious values, and beliefs about a person’s place in the world that may favor or hinder the transition to parenthood, and which are not included in the tool we selected in the validation process.

### Polish version of the Multidimensional Scale of Motives for Postponing Parenthood

While the literature on deferred parenthood is rich in analyses of this topic from a sociological and medical point of view, psychological research is in the minority. In fact, we know little about the motives for deferred parenthood, which are related to the self-perception of the potential parents, their self-focus, understanding of parenthood, fear of changes, financial concerns, or worries about the child’s future. We know even less about the fears, ambivalences, and conflicts surrounding the decision of whether and/or when to have the first child [32]. This indicates that motives for postponed reproductive decision-making is a field that requires precise psychological research that could be replicable and would allow comparisons to be made internationally and cross-culturally.

The analysis concerning the psychological side of the phenomenon of deferring to take on the role of a parent also shows that there are no questionnaires to measure motives for postponing parenthood. This gap seems to be filled by the Multidimensional Scale of Motives for Postponing Parenthood (MSMPP-18) authored by Szcześniak et al. [38]. The MSMPP-18 was developed on the basis of previous narrative studies, statements on blogs devoted to postponed parenthood, and the opinions of emerging adults who do not have children yet. Considering that in many English-speaking countries there is an increase in the age at which women give birth to their first child, as mentioned in the Introduction, an English translation and validation of a questionnaire measuring motives for postponing parenthood seemed necessary.

The original version of the MSMPP-18, designed to assess the motivational forces that may lead to the decision to postpone parenthood, consists of 18 statements grouped into six dimensions that reflect its internal structure: 1) feeling of uncertainty and incompetence refers to the person’s doubts about their own ability to cope with the parental role; 2) self-focus indicates a concentration on one’s personal growth and the realization of one’s goals; 3) parenthood as a burden concerns the perception of parenthood as requiring sacrifice and giving up one’s own aspirations; 4) fear of change is related to the fear of a decline in the quality of the partners’ relationship and unfavorable body changes in women; 5) financial security concerns refer to people’s firm belief that their financial situation does not permit them to give birth and raise a child because of the high cost of living; and 6) worry about a child’s future illustrates people’s concern about potential war, insecure times, and climate change, which could affect the life of their offspring [38; Appendix]. The MSMPP-18 can be treated as a first factor model of six dimensions and a second-order model of the overall dimension of delayed parenthood. Each factor has its own specificity and alludes to motives that may result in postponing the decision to become a parent. Participants declare their personal approach on a 7-point Likert scale from 1 = *I strongly disagree* to 7 = *I strongly agree*.

In the Polish version, the MSMPP-18 [38], both the individual dimensions and the total factor corroborated very good internal reliability. In the process of English-language validation of the MSMPP-18, the first stage was the translation of the original version of the questionnaire from Polish into English by two independent psychologists proficient both in academic Polish and academic English. In the second step, the received translations were consulted with a committee consisting of three bilingual experts experienced in validating psychometric tools and an American psychologist with the experience of teaching in different cultural contexts. Based on their opinions with respect to some linguistic nuances, the best version of the translation was created. In the third step, the scale was subjected to reverse translation to ensure the accuracy of the initial translation. There was a very high consistency between the back translation of the MSMPP-18 and its original version.

The MSMPP-18’s criterion validity in the English-language version of the MSMPP-18 was assessed to expand the existing nomological set of delayed parenthood motives. The justification of the correlational relationships is presented in the section devoted to the aim of Studies 1–3.

### Aim and hypotheses of studies 1–3

**Fig 1:**
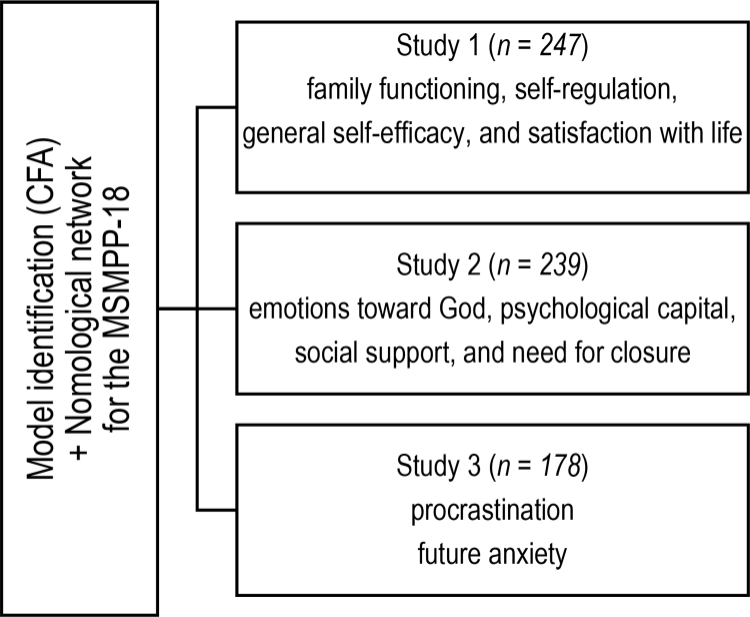
Visual diagram summarizing the overall study design.

Since the MSMPP-18 seems a reliable measure for investigating motives for postponing parenthood, the main goal of Studies 1–3 presented in the current article was to ascertain whether three new datasets with English-speaking participants yield goodness-of-fit indices consistent with the original Polish version. The second aim was to confirm its convergent validity. The stated goals were achieved in three separate studies for two main reasons. First, we wanted to avoid overloading respondents with an excess of questions, thus risking unreliable completion. Second, by spreading the method to other study groups, we were able to test whether the structure of the questionnaire would confirm itself across different samples. Fig 1 presents a visual diagram summarizing the overall study design, thus showing which variables were included at each stage to build a nomological network for the MSMPP-18-EN.

Correlational analyses were conducted according to the presented diagram to check whether and how the motives for postponing parenthood are related to other conceptually similar variables: family functioning, self-regulation, general self-efficacy, and satisfaction with life (Study 1); emotions toward God, psychological capital, social support, and need for closure (Study 2); and procrastination, and future anxiety (Study 3).

#### Study 1.

Previous research yields contradictory findings about postponed parenthood and family functioning [37]. However, the positive correlation between later parenting and experiencing distress in a family context is supported by research on avoidant decision-making in situations of anxiety. In fact, it has been found that an avoidant coping strategy is commonly used when facing a distressing event [39]. If deferred parenthood stems from a fear of one’s own incompetence in caring for a child, the loss of opportunities for self-development, parenthood perceived as a burden, changes in the relationship, lack of sufficient finances, or the child’s future, then it can be expected that it will also be accompanied by family functioning distress.

H1: Motives for postponed parenthood correlate positively with family functioning distress.

The negative relationship between the motives of deferred parenthood and self-regulation is justified by the essence of the latter. A comprehensive review of research on goal-directed behavior shows that self-regulation consists in the capacity of the self to alter and adjust its actions to different social and situational demands [40]. It also includes the ability to delay gratification and make choices across a broad spectrum of behaviors [41]. At the same time, self-regulation failure causes personal problems and significant suffering, which are associated with many phenomena, including procrastination and inaccurate goal setting [42]. People engage in self-regulation activities to align their current lives, their judgments and behaviors with the concept of a “good life,” their ideal self [43]. In people who declare a general desire to have children, but delay the onset of parenthood, self-regulatory processes may be weakened and insufficiently adjusted to the context of their lives (by bringing about conception and childbirth) to realize their life standard, risking a negative balance of life gains and losses [44]. Based on these premises, it can be hypothesized that both variables will be associated negatively.

H2: Motives for postponed parenthood correlate negatively with self-regulation.

The literature emphasizes that delayed parenthood is often related to how potential parents perceive themselves. People who postpone the decision about becoming a parent may feel less competent and have a lower sense of confidence before undertaking the responsibility of raising a family [17]. If parental self-efficacy consists in the ability to successfully carry out tasks related to parenting [45,46], then it can be expected that people who postpone the decision to become a parent may experience a reduced sense of self-efficacy. In fact, low parenting self-efficacy was associated with a propensity to concentrate on difficulties and feelings of helplessness [47].

H3: Motives for postponed parenthood correlate negatively with self-efficacy.

Hypothesis H4 may be justified by research on life cycle theory, the context of decision-making, and procrastination. Garrison et al. [2, p. 282] observed that “according to traditional life cycle theories, parenthood for couples who delay will be less satisfactory and more difficult than for those who do not delay parenthood.” This approach found its confirmation among delayed parents who declared lower marital satisfaction than not-delayed parents [2]. Moreover, several studies confirm that the tendency to avoid decisions or postpone them is negatively related to optimism [48], well-being [49], and job satisfaction [50]. There is also empirical evidence that procrastination, understood as a voluntary delay, predicts lower levels of life satisfaction and subjective well-being [51]. Although these results were verified in groups other than those postponing parenthood, it can be assumed that a similar pattern may also occur in those who procrastinate the decision to have their first child.

H4: Motives for postponed parenthood correlate negatively with life satisfaction.

#### Study 2.

A literature review has shown that the existing studies on delayed parenthood lack the viewpoint of how religion influences reproductive choices and decisions [22,52]. However, there is some evidence that distinct religious populations can be characterized by relatively high fertility [14]. More precisely, some differences in voluntary childlessness can be accounted for by the impact of religious values [53]. It has been found that religion has a stronger effect than education with respect to deliberate childlessness [52]. Women who are religious tend to adhere to religious scripture and traditions that promote pro-natalism and, therefore, have a strong positive effect on entering maternity. Consequently, based on the available results, it can be assumed that positive emotions toward God, which can characterize religious people, will negatively correlate with deferred parenthood.

H5: Motives for postponed parenthood correlate positively with negative emotions toward God and negatively with the positive emotions toward God.

Psychological capital refers to human personal resources that form a high-order construct including: self-efficacy, hope, optimism, and resiliency [54]. Although the components of psychological capital are theoretically different, they share a common variance [55]. People who represent psychological capital and obtain high results in these resources are likely to set higher goals and pursue achieving them [56]. They also have the capacity to cope with adversity [54] and deal with challenging tasks [57]. Taking into account that the motives for deferred parenthood are related to uncertainty and overload, it can be hypothetically assumed that they will correlate negatively with psychological capital.

H6: Motives for postponed parenthood correlate negatively with psychological capital.

In addition to personal capital, social capital in the context of deferred parenthood is also important. According to different studies, social support is an essential resource for family members dealing with stress, playing a protective and buffering role against anxiety [58]. Since childbearing is a source of strain for many young people, a lack of emotional, instrumental, informational, and appraisal support provided by family members, friends, and the larger community can lead to postponed parenthood [4]. Conversely, the implementation of extensive parental support actions and policies may mitigate such parenthood postponement [59].

H7: Motives for postponed parenthood correlate negatively with perceived social support.

The need for closure denotes individuals’ “desire for a firm answer to a question, any firm answer as compared to confusion and/or ambiguity” [60, p. 6]. People with a strong need for closure prefer order and predictability, are relatively closed-minded, feel discomfort with uncertainty and show intolerance of ambiguity [61]. Because uncertainty is at the root of motives for postponing parenthood, people who find it difficult to decide to have their first child may also feel the need for closure. Moreover, both constructs are also positively associated with procrastination, which may indicate that individuals who have difficulty making the decision to start a family run from challenges and ambiguity through procrastination.

H8: Motives for postponed parenthood correlate positively with the need for closure.

#### Study 3.

As Cutas et al. [62] point out, procreative procrastination is a complex social phenomenon that goes beyond the individual responsibility of the mother. Procrastination, however, can only apply to people who originally had the intention of becoming pregnant. There is a group of women who have never discovered such a desire at some point in their lives, in which case there can be no procrastination. Postponed adulthood might be seen as a kind of developmental procrastination [63]. Taking into account the fact that the related concept of postponement of becoming an adult might be either an expression of functional or dysfunctional indecisiveness, we predict that postponed parenthood reveals a significant positive correlation with procrastination [64,65]. In the research by Bańka and Hauziński [63], globalized career indecision was correlated with commitment making, understood as the extent to which a person undertakes long-term life choices and responsibilities in the area of important matters related to identity development. This is supported by other results saying there is a correlation between procrastination and variables measuring significant life decisions. According to research by Tuli [66], procrastination correlates with career anxiety (**r* *= 0.28; *p* ≤ 0.01).

H9: Motives for postponed parenthood correlate positively with procrastination.

Young people may postpone the decision to have their first child due to anxiety about their own insufficient skills in caring for the child, loss of freedom, changes in relationships, or fears about the child’s future and safety [38]. Zaleski [67] explains that future anxiety may refer to concern about not becoming a good parent. Conversely, people who believe they cannot be in control of possible threats engage in anxious thinking. According to Behnagh and Ferrari [68], delay, as a type of avoidance strategy to cope or protect oneself, can be a way to handle one’s anxiety. Therefore, traits combining the motives of deferred parenthood and future anxiety justify hypothesis H10.

H10: Motives for postponed parenthood correlate positively with future anxiety.

## Study 1

Study 1 focuses on a first confirmation (CFA) of the 18-item MSMPP-18-EN and addresses hypotheses H1–H4. Registered hypotheses and data from this project (Studies 1–3), may be found at https://doi.org/10.17605/OSF.IO/TSFB5.

### Method

#### Participants.

The deliberate sample involved 247 childless adults (54% women, 40% men, 6% preferred not to say) who were recruited through a data collection platform (CloudResearch) and took a 12-minute survey. We set initial criteria for recruiting respondents (people aged 18–45, born in the United States, with citizenship and currently residing there, speaking English). The survey was launched on 19/05/2024 at 4:41 PM EST, and ended on 20/05/2024 at 3:19 AM EST. Each of the approved respondents received a completion code on the last page of the survey, which they could redeem for compensation ($2). A total of 321 people took part in the survey. After the survey began, 61 people dropped out, citing, among other reasons, inaccurate knowledge of the nature of the target group (as we recruited childless people). Another 12 people were timed-out (the system automatically excluded them due to possible unreliable completion of the survey). One person who performed the survey twice was rejected. The mean age of the respondents was 32.24 (*SD* = 7.14). The youngest of them were 18, and the oldest were 45. Detailed characteristics of the study participants for Study 1–3 are provided in the Supplementary materials (S1 Table).

A written informed consent form to participate in Studies 1–3 was returned by all respondents. Before starting the research, the project was submitted to the Research Ethics Committee of the Institute of Psychology at the University of Szczecin (No. 24/2023 of 09.11.2023) and received its agreement. The studies were conducted from February to June 2024 according to the ethical principles set out in the Declaration of Helsinki. The analyses in Studies 1–3 were conducted using the IBM SPSS software and IBM SPSS AMOS 21.

#### Procedure and data analysis.

The main aim of Studies 1–3 was to check whether the psychometric structure of the Polish version of the MSMPP-18 is confirmed in the English model.

Before conducting a CFA [69], the data of Studies 1–3 was analyzed for the presence of influential outliers through the Mahalanobis distance (the critical value of χ^2^ with degrees of freedom at **p* *< 0.001) and Cook’s distance (rule of thumb—a value of 0.5 or larger is considered problematic) [70,71]. The variance inflation factor (VIF) and a tolerance value for each independent variable were used as diagnostic tools to quantify collinearity [72]. A VIF above the value 10.0 [73] and a tolerance value equal to 0.1 or less were considered as potentially harmful indicatives of multicollinearity [72]. The model verification was performed using the Confirmatory Factor Analysis (CFA) and its five-step approach: 1) specification; 2) identification; 3) estimation; 4) fit; and 5) model modification.

Model specification was defined by proposing a six-factor structure of the MSMPP-18-EN and aligning the items (observed variables) to each factor based on theoretical and empirical premises [74]. A theoretical rationale of the model was based on the assumption that delaying the decision of having the first child is a complex phenomenon that depends on several factors related to the self (e.g., uncertainty, self-focus, and fear of change) and socio-cultural factors (e.g., parenthood as a burden, financial concern, and worry about the child’s future). The empirical justification was drawn on the structure of the scale obtained in the original study [38], with inclusion of factor loadings, factor variance, factor covariances, residuals, and residual variances. Moreover, given that the Polish version of the scale showed not only a first-order model but also a second-order model, suggesting that the motives for postponing parenthood represent aspects of a more inclusive concept [72], the six factors were used as indicators of the more general construct, which was called postponed parenthood.

Model identification was performed according to several general rules for determining multi-factor CFA models [75]. The three-indicator rule was followed: 1) each latent construct should have at least three observable indicator variables (scale items) with non-zero factor loadings; 2) manifest variables should load on only one factor; 3) the measurement error terms should not be correlated. Model estimation included assessment of model parameters through the maximum likelihood (ML) estimation procedure, which is considered a method robust to violations of normality [76] that is unbiased, consistent, and efficient [77]. Model evaluation consisted of examining the results indicating how well the hypothesized model fit the empirical data [78]. Due to existence of a large set of fit indices to assess the model fit, its adequacy was verified with the use of the multiple fit indices [78,79]: chi-square (χ^2^) with a *p* insignificant value sensitive to the sample size; chi-square/ degrees of freedom ratio (χ^2^/*df*) ≤ 3; Goodness-of-Fit Index, GFI ≥ 0.9; Tucker-Lewis Index, TLI ≥ 0.9; Comparative Fit Index, CFI ≥ 0.9; Root Mean Square Error of Approximation, RMSEA ≤ 0.8, its 90% Confidence Interval with LO ≤ 0.05; HI ≤ 0.08; and Standardized Root Mean Square Residual, SRMR ≤ 0.10 [79,80].

Due to the multiple interpretations regarding convergent validity and the magnitude of the standardized factor loadings (between ≥ 0.4 and at least ≥ 0.7) [81], a value no less than 0.55 was accepted as an indication of relatedness between all indicators and factors assigned to them [82]. The reliability of the internal consistency of the MSMPP-18-EN was estimated using Cronbach’s alpha (α) and its two recommended alternatives [83]: McDonald’s Omega (ω) and composite reliability (CR). All estimates were considered acceptable for research purposes, with values above 0.7 [84].

In case of poor model fit, the use of a model modification was considered through adding correlated measurement errors between indicators of the same factor [85] both on the values of the modification indices and a theoretical justification [86].

To assess construct validity, nomological validity was performed in Studies 1–3. The strength of the linear relationship was checked using Pearson’s correlation with the confidence intervals reported for 5% and 95% (bootstrap sampling analysis 1,000). The correlation coefficients were interpreted based on values acknowledged in the social sciences presented by Walker and Almond [87, p. 156]: little or no association (between ± 0.00 and ± 0.14), weak (between ± 0.15 and ± 0.30), moderate to fairly strong (between ± 0.31 and ± 0.59), strong relationship (between ± 0.60 and ± 1.0). Due to the lack of other tools to measure motives for delaying parenthood, we used questionnaires that seem to be conceptually similar. In Study 1, the nomological variables were as follows: family functioning, self-regulation, self-efficacy, and life satisfaction.

#### Measures.

To verify how motives for postponed parenthood relate to other questionnaires that measure related constructs (family functioning; self-regulation; general self-efficacy; and satisfaction with life) we used:

*Multidimensional Scale of Motives for Postponing Parenthood* (MSMPP-18-EN; [38]) consists of 18 items, divided into 3-item factors. Each sentence begins with the following expression: “I am postponing the decision to have a child because…”. Six factors refer to the motives that may cause postponing the decision to have the first child: 1) “uncertainty and incompetence” (e.g., once’s belief in their incapacity to cope with the role of a parent); 2) self-focus (e.g., self-development); 3) parenthood as a burden (e.g., sacrifice); 4) fear of change (e.g., worsening sexual satisfaction in the relationship); 5) financial security concern (e.g., economic difficulties); 6) worry about the child’s future (e.g., war, climate change) [38]. The respondents express their level of agreement or disagreement on a 7-point Likert scale (1 = *I strongly disagree* and 7 = *I strongly agree*). Higher scores mean greater motives for postponing parenthood. The internal consistency of the total MSMPP-18-EN and its subscales, measured through Cronbach’s alpha (α), McDonald’s Omega (ω), and composite reliability (CR), were very good (Table 1).

**Table 1:** Study 1: *Descriptive Statistics, Skewness, Kurtosis, Standardized Factor Loadings (1*^*st*^*/2*^*nd*^ **Order), Cronbach’s* α/ *McDonald’s Omega* ω/ *Composite Reliability* CR *(N = 247).**

Items	Mean	SD	Min	Max	Skewness	Kurtosis	Standardized factor loadings (1^st^/2^nd^ order) α/ ω/ CR
MPP1	5.89	1.59	1	7	−1.69	2.13	0.75/0.75
MPP2	3.33	2.04	1	7	0.41	−1.11	0.77/0.75
MPP3	2.71	1.93	1	7	0.87	−0.47	0.70/0.68
MPP4	5.78	1.77	1	7	−1.44	0.96	0.90/0.90
MPP5	4.13	2.16	1	7	−0.10	−1.37	0.87/0.87
MPP6	5.06	1.94	1	7	−0.80	−0.52	0.74/0.73
MPP7	4.39	2.10	1	7	−0.32	−1.22	0.92/0.92
MPP8	5.50	1.79	1	7	−1.21	0.44	0.85/0.86
MPP9	3.78	2.19	1	7	0.19	−1.44	0.67/0.66
MPP10	4.09	2.25	1	7	−0.10	−1.44	0.85/0.87
MPP11	5.09	1.90	1	7	−0.75	−0.56	0.92/0.92
MPP12	4.91	2.00	1	7	−0.58	−0.94	0.85/0.85
MPP13	5.73	1.80	1	7	−1.37	0.76	0.95/0.95
MPP14	5.14	1.85	1	7	−0.76	−0.54	0.93/0.93
MPP15	5.85	1.67	1	7	−1.55	1.53	0.84/0.83
MPP16	3.42	2.16	1	7	0.33	−1.26	0.61/0.60
MPP17	3.47	2.08	1	7	0.19	−1.32	0.83/0.84
MPP18	5.71	1.83	1	7	−1.38	0.83	0.96/0.96
UNCERTAINTY	12.29	5.68	3	21	−0.04	−1.13	0.86/ 0.86/ 0.93
SELF_FOCUS	15.12	5.37	3	21	−0.68	−0.66	0.93/ 0.93/ 0.95
BURDEN	17.24	4.46	3	21	−1.44	1.59	0.86/ 0.86/ 0.89
CHANGE	9.59	5.04	3	21	0.34	−0.77	0.75/ 0.76/ 0.91
FINANCE	17.22	5.18	3	21	−1.38	0.89	0.95/ 0.96/ 0.97
WORRY	12.48	5.38	3	21	−0.15	−1.00	0.83/ 0.83/ 0.93
MPP	83.97	20.81	18	126	−0.68	0.30	0.89/ 0.88/ 0.93
BAFFS	6.25	2.33	3	12	0.44	−0.27	0.89/ 0.89/ 0.80
SRS	20.76	4.47	9	28	−0.25	−0.55	0.89/ 0.94/ 0.96
GSE	29.41	5.92	10	40	−0.43	−0.14	0.92/ 0.97/ 0.97
SAT	16.37	8.32	5	35	0.31	−1.04	0.94/ 0.97/ 0.98

Note. MPP—Total Score for Motives for Postponing Parenthood; BAFFS—Family Functioning; SRS—Self-Regulation; GSE—General Self-Efficacy; SAT—Satisfaction with Life.

*Brief Assessment of Family Functioning Scale* (BAFFS; Mansfield et al. [88]) is an ultra-brief (3 items) and robust measure of general family functioning (satisfaction or distress). The respondents assess an overall picture of their family by choosing an answer on a 4-point Likert scale that ranges from 1 = *strongly agree* to 4 = *strongly disagree*. In the original version of the BAFFS, the coefficient alpha was 0.71.

*Self-Regulation Scale* (SRS; [89]) is a short self-report instrument used to measure attention control in goal pursuit when facing difficulties in achieving it [90]. The 7-item scale is assessed using a 4-point Likert scale from 1 = *not at all true* to 4 = *completely true*. The higher the total score, the higher the level of someone’s self-regulation. Cross-cultural studies confirm its good internal consistency being higher than α = 0.73.

*General Self-Efficacy Scale* (GSES; [91]) is a self-report instrument used to measure a general sense of perceived self-efficacy and optimistic self-belief. The one-dimensional scale consists of 10 items. The respondents assess each of the statements by choosing an answer on a 4-point Likert scale that ranges from 1 = *not at all true* to 4 = *exactly true*. The higher the overall score obtained, the higher the individuals’ belief in their own abilities to face novel or challenging situations. The scale has obtained high internal consistency in different samples worldwide (from 0.82 to 0.93).

*Satisfaction with Life Scale* (SWLS; [92]) is a self-report tool used to measure satisfaction with the respondent’s life as a whole. The instrument consists of five items that form one scale. The respondents assess the accuracy of each statement in relation to their life, by using a seven-point Likert scale from 1 = *strongly disagree* to 7 = *strongly agree*. The higher the total score, the higher the level of overall life satisfaction. The SWLS has good reliability, rated at more than 0.8 in a variety of cultural contexts.

### Results

Table 1 shows the descriptive statistics of all items and factors of the MSMPP-18-EN, family functioning (BAFFS), self-regulation (SRS), self-efficacy (GSE), and life satisfaction (SAT).

All VIF values were rather low and ranged from 1.069 to 2.316. The tolerance values were above 0.1 and varied between 0.432 and 0.935. Thus, both results suggest that multicollinearity should not be a serious problem in sample 1. There was no observation with very high residuals (*p* value was equal to 0.001791). The Cook’s distance values were much less than 1 (ranged between 0.000 and 0.048).

Therefore, both outcomes suggest that there were no potential outliers in Sample 1. The linear regression model showed that the sociodemographic variables included in the analysis—sex (β = 0.043, *t* = 0.723, *p* = 0.471), age (β = −0.247, *t* = −4.116, *p* = 0.001), education (β = 0.075, *t* = 1.238, *p* = 0.217), and decision to have a child in the future (β = 0.319, *t* = 5.132, *p* = 0.001)—explained 17.8% of the variance (R^2^ = 0.178). Other variables displayed an additional 13.7% of the variance. Therefore, it can be supposed that age and decision to have a child in the future may be variables that play a significant role in deciding to postpone parenthood.

**Fig 2:**
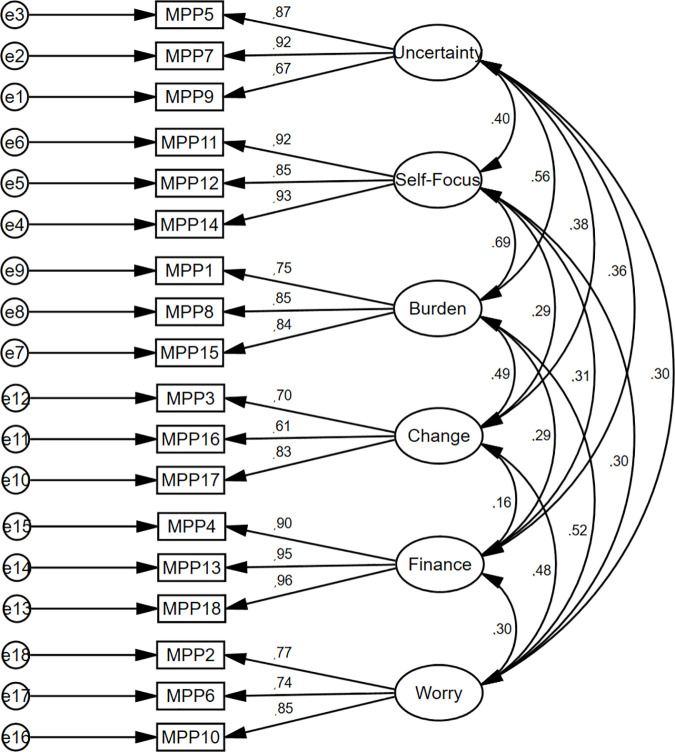
Study 1. Measurement of the 1^st^ order model of the MSMPP-18-EN.

The CFA model was specified based on a six-factor solution of the original version [38]. The factorial structure of the Polish MSMPP-18 was confirmed and its factor loadings were above 0.55 (between 0.61 and 0.96) for all eighteen items (Fig 2).

**Fig 3:**
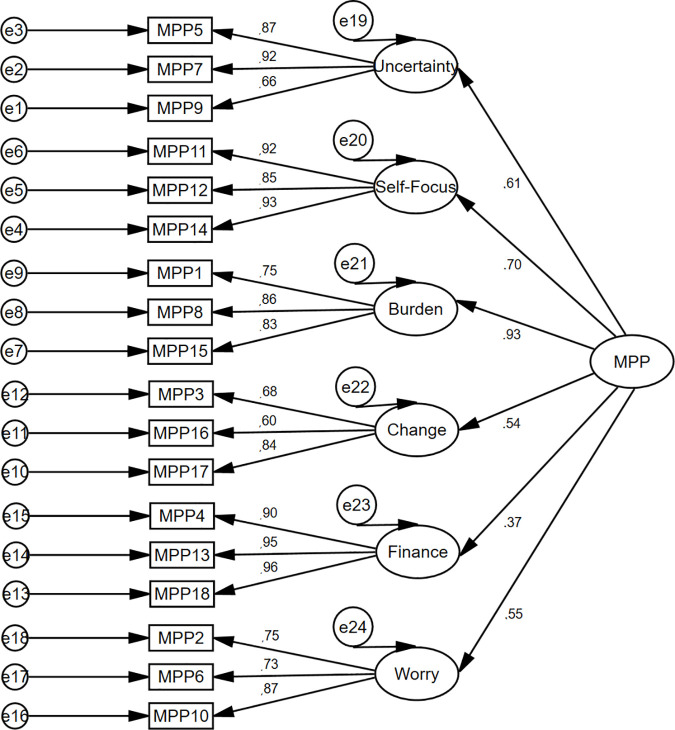
Study 1. Measurement of the 2^nd^ order model of the MSMPP-18-EN.

Moreover, the goodness-of-fit of a six-factor solution presented a very good fit of the model: χ^2^ = 261.225, *p* < 0.001; χ^2^/*df* = 2.177; GFI = 0.90; CFI = 0.95; TLI = 0.94; RMSEA = 0.07, LO = 0.06, HI = 0.08; and SRMS = 0.05. Although χ^2^ was significant, indicating a bad fit, it is generally accepted that this value tends to be statistically significant in groups larger than 200 [93]. Therefore, since the model well represents the sample data, there was no need to identify areas of its misfit through the modification indices. The second-order structure of the MSMPP-18-EN was comparable to its first-order structure (Fig 3), both in terms of factor loadings values (between 0.60 and 0.96) and indices of model fit: χ^2^ = 296.096, *p* < 0.001; χ^2^/*df* = 2.295; GFI = 0.88; CFI = 0.94; TLI = 0.93; RMSEA = 0.07, LO = 0.06, HI = 0.08; and SRMS = 0.07. The linear relationship between the six subscales of the MSMPP-18-EN/its total score and other variables showed various degrees of strength (Table 2).

**Table 2:** Study 1: Correlations between subscales of and total MSMPP-18-EN, family functioning, self-regulation, general self-efficacy, and life satisfaction (N = 247).

	UNC	SFO	BUR	CHA	FIN	WOR	MPP	BAF	SRS	GSE	SAT
UNC	1										
SFO	.363*** [.248;.472]	1									
BUR	.459*** [.358;.552]	.608*** [.496;.700]	1								
CHA	.274*** [.148;.389]	.259*** [.138;.368]	.403*** [.308;.489]	1							
FIN	.375*** [.245;.493]	.295*** [.158;.427]	.266*** [.118;.408]	.117^t^ [-.030;.255]	1						
WOR	.290*** [.168;.409]	.259*** [.123;.392]	.440*** [.334;.537]	.394*** [.283;.502]	.278*** [.141;.407]	1					
MPP	.701*** [.634;.758]	.692*** [.615;.756]	.775*** [.711;.827]	.602*** [.527;.670]	.585*** [.485;.673]	.664*** [.599;.723]	1				
FFU	.177** [.042;.306]	−.010 [-.146;.119]	−.007 [-.146;.134]	−.042 [-.167;.085]	.184** [.047;.310]	.022 [-.115;.162]	.086 [-.048;.217]	1			
SRE	−.334*** [-.446;-.216]	.036 [-.093;.169]	−.093 [-.215;.031]	−.027 [-.159;.100]	−.167** [-.300;-.026]	−.037 [-.178;.103]	−.159* [-.286;-.030]	−.250*** [-.377;-.116]	1		
GSE	−.452*** [-.553;-.344]	−.021 [-.154;.111]	−.121 [-.237;.003]	−.067 [-.196;.066]	−.224*** [-.347;-.094]	−.078 [-.207;.055]	−.247** [-.358;-.129]	−.305*** [-.437;-.172]	.667*** [.577;.749]	1	
SAT	−.299*** [-.408;-.183]	−.164** [-.286;-.033]	−.101 [-.224;.032]	.055 [-.071;.185]	−.353*** [-.471;-.225]	−.061 [-.196;.072]	−.236*** [-.356;-.113]	−.378*** [-.502;-.243]	.295*** [.177;.410]	.484*** [.399;.566]	1

*Note:* ^t^ 0.05 < *p* < 0.1; * *p* < 0.05; ** *p* < 0.01; *** *p* < 0.001; UNC—Uncertainty; SFO—Self-Focus; BUR—Burden; CHA—Change; FIN—Finance; WOR—Worry; MPP—Total score for Motives for Postponing Parenthood: Total; FFU—Family Functioning; SRE—Self Regulation; GSE—General Self-Efficacy; SAT—Satisfaction with Life.

Family functioning (H1) was weakly, positively and significantly associated with uncertainty and finance; the remaining factors were not significantly correlated. Self-regulation (H2) and self-efficacy (H3) correlated statistically significantly, but negatively, with uncertainty, finance, and the total score of the MSMPP-18-EN. Life satisfaction (H4) was negatively associated with uncertainty, self-focus, finance, and the total score of the MSMPP-18-EN. Based on the results obtained, it can be assumed that the four hypotheses in Study 1 were partially confirmed.

### Discussion

The goal of Study 1 was to confirm through CFA the internal structure of the six-dimensional English version of the MSMPP-18. The model evaluation and comparison of fit indices to traditional cut-offs [94] showed a close fit of the structural equation model to the data, thus providing evidence that the American variant of the MSMPP-18 corresponds to its Polish version (both the 1^st^ and the 2^nd^ orders) and does not require any revision or model modification. Moreover, four hypotheses (H1–H4) were examined and largely confirmed in Study 1. In the hypothesis (H1), we expected that a lack of satisfaction with family functioning would correlate positively with the motives for postponed parenthood. The results showed that negative experience of family life (e.g., lack of confidence in each other, not getting along well together) is positively associated with one’s belief in one’s own incapacity to cope with the role of a parent and concern about financial security. These findings can be explained by previous research. For example, Dion [95] confirmed that delayed childbearing is connected to a more individualistic view of family functioning. This suggests that the loosened ties between family members that are characteristic of individualism [96] may be linked to doubts about one’s ability to be a good parent and to financial concerns. Hypothesis (H2) was partially confirmed, as the results showed a negative correlation between self-regulation, uncertainty, financial concern, and overall postponed parenthood. Since self-regulation is a dispositional variable, which refers to any strategy undertaken by individuals to achieve their goals [97] and helps facilitate their effective pursuit [98], it can reduce uncertainties related to one’s own functioning, financial situation, and the decision to postpone parenthood. This result provides evidence that individual differences in self-regulation are important in decision-making in the context of childbearing. It also confirms the theory of planned behavior because both positive and negative intentions to have a child are a function of individuals’ behavioral control [99].

Hypothesis (H3) was partially confirmed, as self-efficacy was negatively associated with uncertainty, financial concern, and overall postponed parenthood. This result is congruent with theoretical premises and empirical evidence. Perceived self-efficacy reflects one’s confidence in their ability to act effectively in the context of prospective situations [100]. When young people do not feel confident, they do not have a sense of preparedness to deal with the challenges of being a parent [17] and are concerned about financial security. Kearney and White [101] found that perceived self-efficacy explained considerable variance in the intention to delay childbearing. In another study, Safdari-Dehcheshmeh et al. [4] suggested that improving self-efficacy can be helpful to improve decision-making in childbearing.

The data from our study supported hypothesis (H4) showing a negative correlation between life satisfaction, uncertainty, self-focus, financial concern, and overall postponed parenthood. Our results are consistent with several previous studies that confirm a negative relationship between well-being and avoidant decision-making [48,49]. Kariman et al. [36] found that quality of life and marital satisfaction were negatively associated with men’s age at the first childbearing decision. A less satisfying relationship with their own mother was associated with being childless at 32 years of age among second-generation Polish and Turkish immigrants [102]. In another study [95], the quality of the relationship with their spouse was one of the predictors of perceived psychological readiness for parenthood.

## Study 2

In Study 2, we again executed a CFA of the MSMPP-18-EN, and addressed hypotheses H5–H8.

### Method

#### Participants.

The purposeful sample consisted of 239 Americans (43.5% women, 52.3% men, 2.9% non-binary; and 1.3%—other) who, like in Study 1, were engaged in the research by means of the Internet through a data collection platform (CloudResearch). We set initial criteria for recruiting subjects that were similar to Study 1. At the same time, the exclusion criterion was participation in Study 1, which allowed us to be sure to obtain data from other individuals. The study was launched on 29/05/2024 at 4:29 PM EST, and ended on 29/05/2024 at 5:29 PM EST. Each respondent whose answers were validated received a completion code, based on which they could collect a sum of compensation ($2). A total of 267 people took part in the survey. After the survey began, 23 people dropped out, citing, among other reasons, a mismatch with their life situation (these people either already had children or did not want children at all). One person was timed-out, and 1 person who took the survey twice was rejected. The participants were between the ages of 18 and 45 (*M* = 31.92; *SD* = 6.82). With respect to political party, 49.8% identified themselves as Democrats; 31.0%—independent; 11.3%—Republicans; 4.6—something else; 3.3%—preferred not to say.

### Measures.

To verify hypotheses H5–H8, we measured the associations between the motives for postponed parenthood, positive and negative emotions toward God, psychological capital, perceived social support, and need for closure using the following scales:

*Emotions towards God Scale* (ETG; [103,104]) is a self-report instrument used to measure negative and positive emotions associated with God. The scale consists of 12 items; 6 of them address positive emotions (e.g., love, security), 5 of them refer to negative emotions (e.g., guilt, punishment), and 1 item reflects disinterest in God. The respondents assess each of the ten statements by choosing an answer on a 5-point Likert scale that ranges from 1 = *does not apply at all* to 5 = *definitely applies*. In its original version, both subscales have good internal reliability (α_positive emotions_ = 0.95; α_negative emotions_ = 0.85).

*Revised Compound Psychological Capital Scale* (CPC-12R; [105]) is a self-report instrument used to measure the core psychological factor of positivity that helps manage tough situations. The scale consists of 12 items forming four components: hope (ability to pursue and achieve goals through motivation and appropriate pathways), self-efficacy (mobilization of one’s own cognitive and motivational resources to increase performance), resilience (ability to deal with and overcome negative experiences), and optimism (capacity to attribute internal factors to positive events, and external causes for negative situations). The respondents assess each of the statements by choosing an answer on a 6-point Likert scale that ranges from 1 = *strongly disagree* to 6 = *strongly agree*. The original version of the CPC-12R revealed very good reliability (α = 0.90).

*Multidimensional Scale of Perceived Social Support* (MSPSS; [106]) is a self-report tool designed to measure the perceived comfort, caring, esteem, or instrumental help a person receives from others. The scale consists of 12 items forming three factors reflecting the perceived adequacy of support from: family, friends, and a significant other. Respondents indicate their answers on a 7-point Likert scale ranging from 1 = *very strongly disagree* to 7 = *very strongly agree*. In the original studies, the coefficient alphas for the three subscales and overall score ranged from 0.72 to 0.85.

*Need for Closure Scale* (NFCS–SV; [107,108]) is a short self-report instrument (15 items) that measures the degree to which a person has a desire for certainty. The NFCS–SV forms one factor. The respondents assess each of the statements by choosing an answer on a 6-point Likert scale that ranges from 1 = *strongly disagree* to 6 = *strongly agree*. High scores on this scale mean that a person values order, feels distaste for ambiguity, decides quickly, and forms strong opinions. In its original version, the 15-item scale has an adequate internal consistency (α = 0.76).

The procedure and data analysis were aimed at verifying the psychometric structure of the MSMPP-18-EN and correlations between motives for postponing parenthood, emotions toward God, psychological capital, social support, and need for closure.

### Results

Table 3 shows the descriptive statistics of all items and factors of the MSMPP-18-EN, positive and negative emotions toward God, psychological capital, perceived social support, and need for closure, and suggests that the distributions of the items and variables are close to a normal distribution.

**Table 3:** Study 2: *Descriptive Statistics, Skewness, Kurtosis, Standardized Factor Loadings (1*^*st*^*/2*^*nd*^ **Order), Cronbach’s* α/ *McDonald’s Omega* ω/ *Composite Reliability* CR *(N = 239).**

Items	Mean	SD	Min	Max	Skewness	Kurtosis	Standardized factor loadings (1^st^/2^nd^ order) α/ ω/ CR
MPP1	5.74	1.56	1	7	−1.52	1.88	0.73/0.73
MPP2	3.24	1.95	1	7	0.44	−1.00	0.79/0.79
MPP3	2.77	1.86	1	7	0.83	−0.47	0.69/0.68
MPP4	5.48	1.83	1	7	−1.13	0.19	0.87/0.87
MPP5	3.73	2.19	1	7	0.17	−1.41	0.87/0.87
MPP6	4.80	1.87	1	7	−0.51	−0.75	0.76/0.76
MPP7	4.14	2.15	1	7	−0.13	−1.36	0.87/0.87
MPP8	5.28	1.87	1	7	−0.95	−0.22	0.72/0.72
MPP9	3.60	2.15	1	7	0.28	−1.33	0.72/0.72
MPP10	3.96	2.12	1	7	−0.03	−1.32	0.90/0.91
MPP11	5.10	1.87	1	7	−0.82	−0.38	0.89/0.89
MPP12	5.11	1.83	1	7	−0.76	−0.43	0.90/0.91
MPP13	5.52	1.74	1	7	−1.00	−0.01	0.91/0.92
MPP14	5.19	1.70	1	7	−0.83	−0.03	0.93/0.93
MPP15	5.72	1.67	1	7	−1.39	1.09	0.85/0.84
MPP16	3.63	2.07	1	7	0.18	−1.21	0.66/0.67
MPP17	3.24	1.96	1	7	0.36	−1.12	0.89/0.88
MPP18	5.41	1.88	1	7	−1.03	−0.05	0.93/0.92
UNCERTAINTY	11.47	5.96	3	21	0.09	−1.29	0.91/ 0.91/ 0.86
SELF_FOCUS	15.40	4.96	3	21	−0.86	0.06	0.91/ 0.91/ 0.93
BURDEN	16.74	4.48	3	21	−1.28	1.19	0.85/ 0.85/ 0.81
CHANGE	9.64	4.61	3	21	0.33	−0.58	0.68/ 0.73/ 0.80
FINANCE	16.40	5.12	3	21	−1.00	0.05	0.93/ 0.93/ 0.93
WORRY	12.00	5.00	3	21	0.04	−0.86	0.74/ 0.81/ 0.86
MPP	81.67	20.02	20	126	−0.31	−0.25	0.89/ 0.89/ 0.99
EG_P	9.89	9.11	0	24	0.17	−1.56	0.95/ 0.97/ 0.96
EG_N	5.53	5.63	0	23	0.84	−0.22	0.81/ 0.84/ 0.99
PC_H	11.73	3.80	3	18	−0.39	−0.44	0.84/ 0.85/ 0.85
PC_E	12.33	3.49	3	18	−0.46	−0.05	0.85/ 0.86/ 0.85
PC_R	11.70	3.72	3	18	−0.37	−0.42	0.83/ 0.83/ 0.83
PC_O	12.94	4.03	3	18	−0.76	−0.08	0.93/ 0.93/ 0.94
PC_T	48.72	13.56	12	72	−0.48	−0.02	0.95/ 0.95/ 0.99
PPS_SO	20.17	7.53	4	28	−0.83	−0.37	0.97/ 0.97/ 0.98
PPS_FAM	19.24	7.28	4	28	−0.69	−0.61	0.95/ 0.95/ 0.96
PPS_FR	18.76	6.99	4	28	−0.54	−0.60	0.95/ 0.95/ 0.97
PPS_T	58.18	18.06	12	84	−0.70	−0.05	0.94/ 0.94/ 0.99
NFC	58.39	13.18	16	90	−0.31	0.10	0.89/ 0.89/ 0.98

Note. MPP—Total score for Motives for Postponing Parenthood; EG_P—Emotions toward God_Positive; EG_N—Emotions toward God_Negative; PC_H—Psychological Capital_Hope; PC_E—Self-Efficacy; PC_R—Resilience; PC_O—Optimism; PC_T—Total; PPS_SO—Perceived Social Support_Significant Others; PPS_FAM—Family; PPS_FR—Friends; PPS_T—Total; NFC—Need for Closure.

It can be seen that all VIF values ranged from 1.069 to 2.101, which is under the threshold of 10.0 that indicates multicollinearity. The tolerance values were above 0.1 and varied between 0.476 and 0.935, proving that no multicollinearity exists among the variables in sample 2. Based on the Mahalanobis distance, we detected only one outlier with the *p* value *p* = 0.0001. Moreover, the Cook’s distance values ranged between 0.000 and 0.048, being much less than 1. The linear regression model showed that sex (β = 0.001, *t* = 0.001, *p* = 1.000), age (β = −0.287, *t* = −4.861, *p* = 0.001), religious beliefs (β = −0.077, *t* = −1.249, *p* = 0.213), attitudes to abortion (β = 0.044, *t* = 0.696, *p* = 0.487), decision to have a child in the future (β = 0.288, *t* = 4.832, *p* = 0.001), ethnicity (β = 0.110, *t* = 1.918, *p* = 0.056), adverse childhood experience (β = 0.090, *t* = 1.554, *p* = 0.121), household income (β = 0.040, *t* = 0.701, *p* = 0.484), marital status (β = 0.038, *t* = 0.658, *p* = 0.511), and political party (β = −0.005, *t* = −0.073, *p* = 0.942) explained 26% of the variance (R^2^ = 0.260). Other variables represented a significant amount of the variance (additional 9.4%) despite controlling for the confounding effects. Based on the results obtained, it can be assumed that age and decision to have a child in the future may be variables whose presence is of great importance in the relationship among the motives for postponed parenthood, emotions toward God, psychological capital, perceived social support, and need for closure.

**Fig 4:**
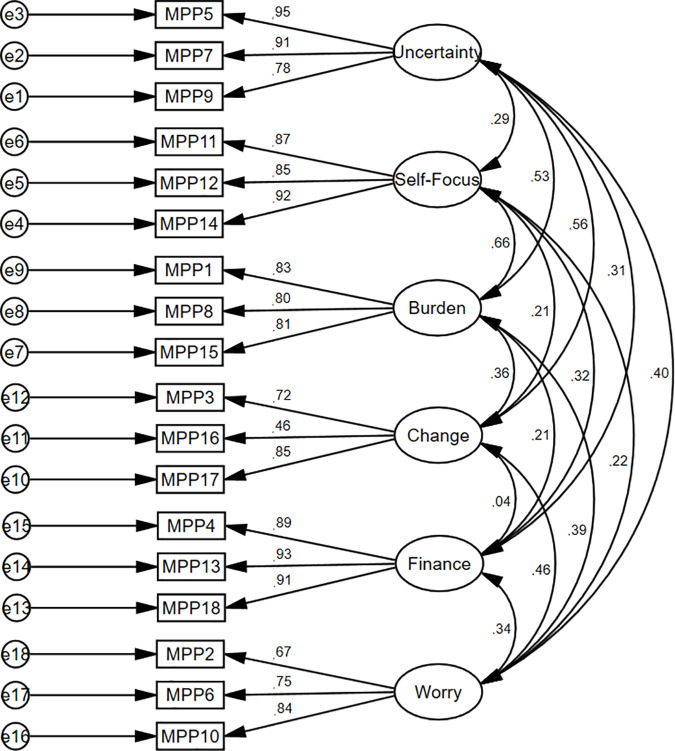
Study 2. Measurement of the 1^st^ order model of the MSMPP-18-EN.

**Fig 5:**
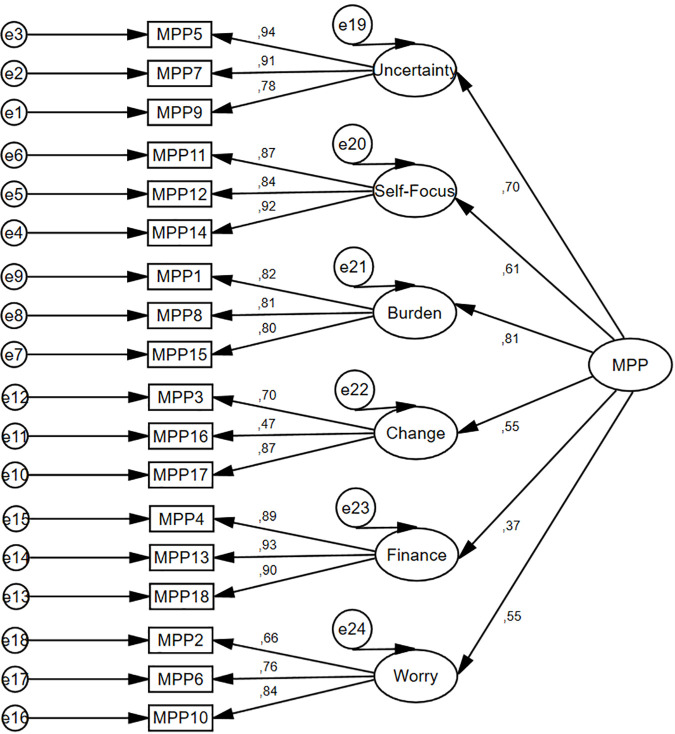
Study 2. Measurement of the 2^nd^ order model of the MSMPP-18-EN.

The factorial structure of the MSMPP-18-EN was confirmed in Study 2. The factor loadings were above 0.55 (between 0.67 and 0.95) for all items of the MSMPP-18-EN except item MPP16 (Figs 4 and 5).

The goodness-of-fit of a six-factor solution presented an acceptable fit of the model: χ^2^ = 214.61, *p* < 0.001; χ^2^/*df* = 1.788; GFI = 0.91; CFI = 0.97; TLI = 0.96; RMSEA = 0.06, LO = 0.05, HI = 0.07; and SRMS = 0.07. As in Study 1, χ^2^ was significant. Moreover, the goodness-of-fit of the second order solution also presented an acceptable fit of the model: χ^2^ = 303.51, *p* < 0.001; χ^2^/*df* = 2.353; GFI = 0.87; CFI = 0.94; TLI = 0.92; RMSEA = 0.08, LO = 0.06, HI = 0.09; and SRMS = 0.10. As in the previous statistics, χ^2^ was significant.

The correlation analysis between the six subscales of the MSMPP-18-EN showed a similar pattern of results as in Study 1 (Table 4). Positive emotions toward God correlated negatively and significantly with motives of uncertainty, burden, financial concern, worry (at the level of tendency), and postponed parenthood overall (H5). Negative emotions toward God correlated positively and significantly with uncertainty, fear of change, worry (at the level of tendency), and overall postponed parenthood. Psychological capital, both in its dimensions and total score, was negatively linked to uncertainty, financial concern, worry, and overall delayed parenthood (H6). Similar results were obtained in the case of perceived social support.

**Table 4:** *Study 2: Correlations (with Confidence Intervals) between Dimensions of MSMPP-18-EN,* ETG, CPC-12R, MSPSS, and NFCS–SV *(N = 239).*

	UNC	SFO	BUR	CHA	FIN	WOR	MPP	EG_P	EG_N	PC_H	PC_E	PC_R	PC_O	PC_T	PS_SO	PS_FA	PS_FR	PS_T	NFC
UNC	1																		
SFO	.286*** [.154;.409]	1																	
BUR	.484*** [.390;.573]	.578*** [.446;.683]	1																
CHA	.499*** [.390;.595]	.243*** [.113;.358]	.376*** [.266;.472]	1															
FIN	.294*** [.160;.415]	.282*** [.154;.409]	.180** [.057;.313]	.070 [-.048;.179]	1														
WOR	.382*** [.253;.501]	.189** [.037;.336]	.326*** [.177;.465]	.429*** [.311;.533]	.290*** [.168;.400]	1													
MPP	.763*** [.708;.811]	.638*** [.535;.723]	.725*** [.652;.788]	.649*** [.563;.720]	.542*** [.450;.624]	.656*** [.562;.736]	1												
EG_P	−.303*** [-.420;-.181]	−.071 [-.210;.063]	−.227*** [-.353;-.098]	−.051 [-.173;.076]	−.208** [-.341;-.076]	−.104^t^ [-.236;.027]	−.250*** [-.372;-.128]	1											
EG_N	.240*** [.119;.356]	.000 [-.126;.126]	−.064 [-.181;.053]	.251*** [.132;.367]	.021 [-.093;.130]	.130* [.009;.247]	.153* [.035;.271]	.279*** [.161;.394]	1										
PC_H	−.342*** [-.466;-.212]	.062 [-.082;.203]	.068 [-.082;.214]	.015 [-.127;.152]	−.396*** [-.488;-.296]	−.183** [-.313;-.046]	−.215** [-.350;-.074]	.306*** [.184;.426]	−.135* [-.255;-.015]	1									
PC_E	−.400*** [-.513;-.277]	.060 [-.085;.207]	.039 [-.100;.179]	−.068 [-.200;.066]	−.344*** [-.445;-.234]	−.244*** [-.369;-.105]	−.260*** [-.384;-.130]	.277*** [.153;.399]	−.210** [-.330;-.094]	.827*** [.772;.870]	1								
PC_R	−.444*** [-.552;-.333]	.037 [-.102;.174]	−.064 [-.190;.062]	−.082 [-.220;.057]	−.349*** [-.453;-.235]	−.137* [-.267;-.007]	−.280*** [-.405;-.151]	.355*** [.235;.466]	−.126* [-248;-.002]	.748*** [.668;.814]	.784*** [.718;.837]	1							
PC_O	−.367*** [-.490;-.241]	.171** [.012;.319]	.080 [0.086;.237]	−.049 [-.187;.083]	−.276*** [-.372;-.173]	−.273*** [-.406;-.127]	−.199** [-.341;-.055]	.263*** [.136;.387]	−.172 [-.283;-.063]	.776*** [.705;.837]	.735*** [.652;.805]	.631*** [.522;.725]	1						
PC_T	−.430*** [-.543;-.312]	.094 [-.055;.242]	.036 [-.108;.179]	−.050 [-.189;.085]	−.378*** [-.476;-.272]	−.233*** [-.362;-.094]	−.263*** [-.394;-.129]	.333*** [.208;.450]	−.178** [-.291;-.063]	.930*** [.906;.948]	.923*** [.895;.945]	.874*** [.829;.911]	.878*** [.840;.911]	1					
PS_SO	−.124^t^ [-.270;.024]	.017 [-.125;.159]	.008 [-.143;.156]	.059 [-.057;.174]	−.183** [-.291;-.064]	−.076 [-.219;.067]	−.083 [-.220;.067]	.181** [.054;.298]	−.002 [-.126;.115]	.406*** [.283;.517]	.323*** [.183;.456]	.277*** [.134;.412]	.477*** [.338;.604]	.415*** [.280;.542]	1				
PS_FA	−.299*** [-.421;-.171]	.039 [-.098;.179]	−.005 [-.154;.139]	−.175** [-.302;-.041]	−.254*** [-.355;-.144]	−.322*** [-.442;-.188]	−.267*** [-.391;-.130]	.241*** [.115;.364]	−0.070 [-.190;.046]	.437*** [.316;.544]	.405*** [.277;.522]	.374*** [.244;497]	.550*** [.431;.657]	.493*** [.378;.598]	.470*** [.337;.595]	1			
PS_FR	−.229*** [-.353;-.102]	.058 [-.097;.202]	.053 [-.093;.190]	−.092 [-.222;.041]	−.285*** [-.385;-.180]	−.215** [-.345;-.078]	−.190** [-.321;-.056]	.118^t^ [-.019;.251]	−.067 [-.201;.064]	.555*** [.443;.652]	.507*** [.389;.609]	.491*** [.367;.600]	.526*** [.401;.636]	.577*** [.468;.672]	.504*** [.378;.635]	.617*** [.498;.724]	1		
PS_T	−.261*** [-.381;-.133]	.045 [-.099;.189]	.022 [-.128;.170]	−.082 [-.198;.039]	−.289*** [-.384;-.182]	−.244*** [-.373;-.107]	−.216** [-.340;-.083]	−.219** [.085;.345]	−.055 [-.184;.066]	.560*** [.454;.652]	.494*** [.370;.605]	.456*** [.329;.568]	.624*** [.515;.718]	.596*** [.487;.689]	.802*** [.742;.856]	.838*** [.786;.882]	.846*** [.800;.886]	1	
NFC	.244*** [.115;.368]	.136* [-.011;.284]	.205** [.057;.353]	.115^t^ [-.018;.247]	.218** [.090;.343]	.178** [.052;.295]	.279*** [.148;.409]	.001 [-.121;.127]	.107^t^ [-.013;.229]	−.163* [-.307;-.103]	−.246*** [-.377;-.103]	−.291*** [-.416;-.154]	−.069 [-.203;.079]	−.209** [-.347;-.060]	.029 [-.109;.171]	−.086 [-.222;.051]	−.197** [-.321;-.065]	−.099 [-.231;.043]	1

*Note:* ^t^ 0.05 < *p* < 0.1; * *p* < 0.05; ** *p* < 0.01; *** *p* < 0.001; UNC—Uncertainty; SFO—Self-Focus; BUR—Burden; CHA—Change; FIN—Finance; WOR—Worry; MPP—Total score for Motives for Postponing Parenthood: Total; EG_P—Emotions toward God_Positive; EG_N—Emotions toward God_Negative; PC_H—Psychological Capital_Hope; PC_E—Self-Efficacy; PC_R—Resilience; PC_O—Optimism; PC_T—Total; PS_SO—Perceived Social Support_Significant Others; PS_FA—Family; PS_FR—Friends; PS_T—Total; NFC_Need for Closure.

Dimensions of significant others, family, friends, and total support correlated negatively with uncertainty, financial concern, worry (except significant others), and postponed parenthood (except significant others) (H7). Need for closure was positively and significantly associated with all the dimensions of postponed parenthood and its total score, except fear of change, which correlated at the level of tendency (H8). Based on the findings obtained in Study 2, it can be suggested that all hypotheses tested in this study (H5–H8) were partially confirmed.

### Discussion

Similarly to Study 1, the main aim of Study 2 was to verify the internal structure of the English version of the MSMPP-18. The model evaluation displayed an adequate fit of the structural equation model to the data both in the 1^st^ order model and the 2^nd^ order model. Moreover, the four hypotheses (H5–H8) were largely confirmed.

With respect to hypothesis H5, a negative correlation between positive emotions toward God and motives of uncertainty, burden, financial concern, worry, postponed parenthood overall, and a positive correlation between negative emotions toward God and motives of uncertainty, fear of change, worry, and overall postponed parenthood are consistent with some previous studies. Such a relationship can be explained by research on religiosity and general or family values. It has been found that religiosity is positively associated with conservative values (tradition and conformity) and negatively with hedonism and self-directed values [109]. Moreover, family behaviors (e.g., marital stability, attitudes toward cohabitation and voluntary childlessness) are related to religious beliefs [110–112]. For example, religious people who attend church at least once a month disfavor volitional childlessness [113]. Individuals who strongly value tradition and declare their religiosity tend to be less favorable to the active choice not to have children [111]. In another study [113], religion was a strong correlate of one’s own childlessness intentions. Although this study is not about deferred parenthood, it concerns a phenomenon of decision-making in the context of having or not having children.

In line with hypothesis H6, dimensions and overall psychological capital (consisting in hope, self-esteem, resilience, and optimism) correlated negatively with uncertainty, self-focus, financial concern, worry, and postponed parenthood. It can be assumed that people who have the capacity to cope with adversity, in other words, have psychological capital, do not tend to postpone parenthood. Empirical confirmation of this hypothesis comes from research on the relationship between dimensions of psychological capital and variables related to parenting decisions. For example, Łada-Maśko and Kaźmierczak [114] suggest that resilience, which alludes to one’s capacity to “bounce back” from adversity to attain success, is an important facet of maturity to parenthood. In other studies, self-efficacy was considered an aspect of psychological preparation for better decision-making in childbearing [115]. Enhancing perceived self-efficacy through reproductive knowledge may encourage women to modify their attitudes toward childbearing [4]. Postponed parenthood seems to be related to hope, as well. In their study, Bodin et al. [116] suggest that some men postpone becoming fathers until a later time because they hope to mature and provide better conditions for raising the child. Finally, Lebano and Jamieson [117], in their qualitative study, showed that people who defer the decision to have children are encouraged to do so by optimism about their capacity to bear a child.

Regarding hypothesis H7, perceived social support, especially from family and friends, correlated negatively with uncertainty, fear of change, financial concern, worry, and postponed parenthood. The obtained results confirm previous research on social capital that may influence women’s decisions regarding postponed parenthood [103]. Since giving birth is an important source of significant psychological distress for many women, a shortage of emotional and instrumental social support from family members, partner, and peers may translate into deferred parenthood [4]. This pattern of correlations can also be explained based on the theory of planned behavior. According to Ajzen and Klobas [118], the decision to have a child depends on positive or negative attitudes toward having children, more or less favorable subjective norms, and greater or weaker perceptions of control in relation to having a child. Moreover, empirical evidence shows that people who declare social support are likely to enter parenthood earlier than their peers who do not disclose this [119]. In fact, the timing of family formation may depend on the attitudes of “others.” For example, potential parents differ in the timing of the decision to have a child, depending on their own parents’ vision of parenthood. If young adults’ mothers prefer that their children engage in later marriages, they tend to have first child later than those adults whose mothers prefer early marriages [119].

Hypothesis H8 found its confirmation as need for closure was positively associated with all the motives for postponed parenthood. This result is not surprising if we take into account that need for closure appears in effortful, costly, and unpleasant circumstances [107]. People with a high level of need for closure prefer order and structure, make decisions quickly, show discomfort caused by ambiguity or uncertainty, and long for secure, predictable, and stable knowledge. On the other hand, the prospect of starting a family can evoke feelings of anxiety, worry, and threat in young people [120] because the presence of a child requires changes in their lives. Therefore, high results in need for closure may elicit the motives of postponed parenthood.

## Study 3

Study 3, similarly to Studies 1–2, was aimed at verifying the psychometric structure of the MSMPP-18-EN. It was also focused on checking correlations between motives for postponing parenthood, procrastination, and future anxiety.

### Method

#### Participants.

The purposeful sample included 178 emerging adults (63.5% women, 35.4% men, 1.1% did not provide an answer). Data was collected via the Internet. The participants were aged between 18 and 45 (*M* = 25.42; *SD* = 5.80).

#### Measures.

In the present study of the MSMPP-18-EN, the Pure Procrastination Scale and the Dark Future Scale were used to verify hypotheses H9–H10 and associations between motives for postponed parenthood, procrastination, and future anxiety.

*Pure Procrastination Scale* (PPS; [121]) is a self-report short tool used to measure the act of delaying or putting off tasks until the last minute or past their deadline. The one-dimensional scale consists of 12 items. Respondents indicate their answers to the question items on a 7-point Likert Scale ranging from 1 = *strongly disagree* to 7 = *strongly agree*. The higher the total score, the more intense the respondent’s procrastination. The reliability of the scale in other studies was determined to be very good (α = 0.82).

*Dark Future Scale* (DFS; [122]) is a short self-report instrument used to measure the tendency to think about the distant rather than the proximate future with anxiety and uncertainty and to anticipate disasters in the future. The scale consists of five items. The respondents assess each of the statements by choosing an answer on a 7-point Likert scale that ranges from 0 = *decidedly false* to 6 = *decidedly true*. The scale is characterized by very good reliability (α = 0.90).

### Results

Table 5 shows the descriptive statistics of all items and factors of the MSMPP-18-EN, procrastination, and future anxiety (dark future). All VIF values ranged from 1.028 to 1.314, being lower than the threshold of 10.0.

**Table 5:** Study 3: *Descriptive Statistics, Skewness, Kurtosis, Standardized Factor Loadings (1*^*st*^*/2*^*nd*^ **Order), Cronbach’s* α/ *McDonald’s Omega* ω/ *Composite Reliability* CR *(N = 178).**

Items	M	SD	Min	Max	Skewness	Kurtosis	Standardized factor loadings (1^st^/2^nd^ order) α/ ω/ CR
MPP1	5.47	1.87	1	7	−1.21	0.27	0.73/0.73
MPP2	3.35	1.92	1	7	0.31	−1.00	0.79/0.79
MPP3	2.70	1.68	1	7	0.82	−0.32	0.69/0.68
MPP4	5.53	1.99	1	7	−1.16	−0.02	0.87/0.87
MPP5	3.16	1.96	1	7	0.59	−0.77	0.87/0.87
MPP6	4.41	2.14	1	7	−0.31	−1.31	0.76/0.76
MPP7	3.40	1.97	1	7	0.31	−1.12	0.87/0.87
MPP8	4.53	2.00	1	7	−0.41	−1.12	0.72/0.72
MPP9	2.96	1.90	1	7	0.70	−0.73	0.72/0.72
MPP10	3.63	2.22	1	7	0.18	−1.37	0.90/0.91
MPP11	5.38	1.78	1	7	−1.10	0.35	0.89/0.89
MPP12	5.43	1.80	1	7	−1.06	0.14	0.90/0.91
MPP13	5.17	2.07	1	7	−0.90	−0.56	0.91/0.92
MPP14	5.40	2.74	1	7	−1.10	0.30	0.93/0.93
MPP15	5.57	1.81	1	7	−1.24	0.44	0.85/0.84
MPP16	3.61	2.02	1	7	0.18	−1.20	0.66/0.67
MPP17	3.04	1.85	1	7	0.56	−0.86	0.89/0.88
MPP18	5.13	2.10	1	7	−0.85	−0.69	0.93/0.92
UNCERTAINTY	9.51	5.15	3	21	0.45	−0.78	0.86/ 0.86/ 0.86
SELF_FOCUS	16.21	5.00	3	21	−1.16	0.56	0.93/ 0.93/ 0.93
BURDEN	15.57	4.83	3	21	−0.98	0.14	0.81/ 0.81/ 0.81
CHANGE	9.35	4.60	3	21	0.31	−0.80	0.77/ 0.79/ 0.78
FINANCE	15.82	5.78	3	21	−0.96	−0.38	0.93/ 0.93/ 0.93
WORRY	11.39	5.53	3	21	0.03	−1.18	0.85/ 0.86/ 0.86
MPP	77.86	22.80	20	126	−0.74	0.15	0.92/ 0.92/ 0.99
PPS	43.13	16.10	12	81	0.01	−0.63	0.95/ 0.97/ 0.98
DFS	17.97	7.18	5	30	−0.09	−1.03	0.81/ 0.84/ 0.96

Note. MPP—Total score for Motives for Postponing Parenthood; PPS—Pure Procrastination Scale; DFS—Dark Future Scale.

The tolerance values were above 0.1 and varied between 0.761 and 0.972. Thus, both results denote the absence of multicollinearity in sample 3. There were no outliers, as all cases had values of *p* > 0.001 (0.002191). The Cook’s distance values were much less than 1 (ranged between 0.000 and 0.102), confirming the absence of outliers in Study 3. The linear regression model showed that sex (β = −0.110, *t* = −1.873, *p* = 0.063), age (β = −0.270, *t* = −4.294, *p* = 0.001), ethnicity (β = 0.080, *t* = 1.356, *p* = 0.177), and decision to have a child (β = 0.303, *t* = 4.982, *p* = 0.001) explained 25.7% of the variance (R^2^ = 0.257). Procrastination and future anxiety represented a significant amount of the variance (additional 16.7%) despite controlling for the confounding effects. Based on the results obtained, it can be assumed that age and decision to have a child may be the variables whose presence is of great importance in the relationship among the motives for postponed parenthood, procrastination, and future anxiety.

**Fig 6:**
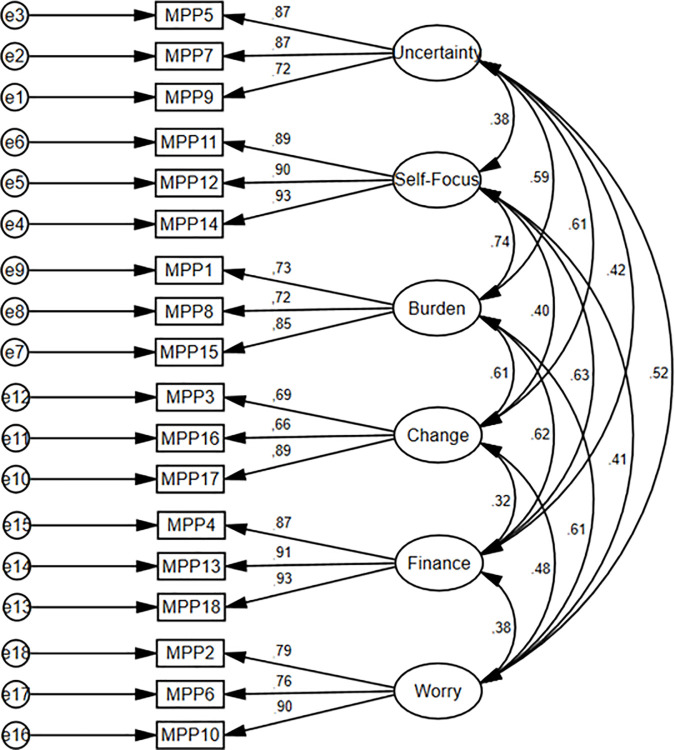
Study 3. Measurement of the 1^st^ order model of the MSMPP-18-EN.

The CFA model was specified based on a six-factor solution. The factorial structure of the MSMPP-18-EN (first order) was again confirmed in Study 3. The factor loadings were above 0.55 for all eighteen items of the MSMPP-18-EN (Fig 6).

**Fig 7:**
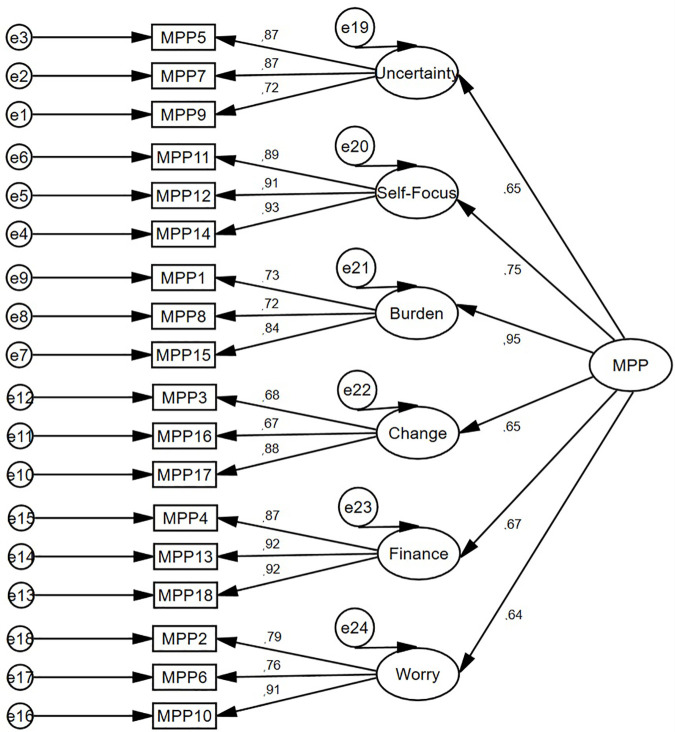
Study 3. Measurement of the 2^nd^ order model of the MSMPP-18-EN.

Moreover, the goodness-of-fit of the model presented an optimal fit: χ^2^ = 201.637, *p* < 0.001; χ^2^/*df* = 1.680; GFI = 0.89; CFI = 0.96; TLI = 0.95; RMSEA = 0.06, LO = 0.05, HI = 0.08; and SRMS = 0.05. Only χ^2^ was significant. The CFA model was specified based on a six-factor solution. The factorial structure of the MSMPP-18-EN (second order) was again confirmed in Study 3. The factor loadings were above 0.55 for all eighteen items of the MSMPP-18-EN (Fig 7). Moreover, the goodness-of-fit of a six-factor solution presented an optimal fit of the model: χ^2^ = 242.692, *p* < 0.001; χ^2^/*df* = 1.881; GFI = 0.86; CFI = 0.95; TLI = 0.94; RMSEA = 0.07, LO = 0.06, HI = 0.08; and SRMS = 0.07.

A summary of the data fit results for each model is presented in Table 6. Looking at the obtained reliability results, a certain regularity occurring in the three studies can be noticed. The factor with the lowest, although still satisfactory, reliability values was fear of change. In turn, the factors with the highest reliability were financial security concern and self-focus.

**Table 6:** Summary of fit indices for Studies 1–3.

	Study 1 (*N = 247*)		Study 2 (*N = 239*)		Study 3 (*N = 178*)	
Fit indices	Six factor solution	Second order solution	Six factor solution	Second order solution	Six factor solution	Second order solution
χ^2^	261.225***	296.096***	214.61***	303.51***	201.637***	242.692***
χ^2^/*df*	2.177	2.295	1.788	2.353	1.680	1.881
GFI	0.90	0.88	0.91	0.87	0.89	0.86
CFI	0.95	0.94	0.97	0.94	0.96	0.95
TLI	0.94	0.93	0.96	0.92	0.95	0.94
RMSEA	0.07	0.07	0.06	0.08	0.06	0.07
LO	0.06	0.06	0.05	0.06	0.05	0.06
HI	0.08	0.08	0.07	0.09	0.08	0.08
SRMS	0.05	0.07	0.07	0.10	0.05	0.07

*** *p* < 0.001.

The correlation analysis between the six subscales of the MSMPP-18-EN showed positive, weak, or moderate, significant relationships (Table 7). Procrastination was significantly and positively associated with all six motives for postponed parenthood and their overall score. The subscales and total score of the motives for postponed parenthood correlated positively and significantly with future anxiety, as well.

**Table 7:** Study 3: Correlations between subscales of MSMPP-18-EN and MPP, PPS, and DFS (N = 178).

	UNC	S-FOC	BURD	CHA	FIN	WOR	MPP	PPS	DFS
UNC	1								
S-FOC	.341*** [.201;.481]	1							
BURD	.517*** [.390;.645]	.622*** [.505;.738]	1						
CHA	.491*** [.362;.621]	.388*** [.251;.525]	.547*** [.423;.672]	1					
FIN	.392*** [.255;.529]	.590*** [.470;.710]	.523*** [.397;.650]	.282*** [.139;.424]	1				
WOR	.455*** [.322;.587]	.378*** [.240;.515]	.520*** [.393;.647]	.436*** [.302;.570]	.352*** [.213;.492]	1			
MPP	.719*** [.616;.823]	.748*** [.649;.846]	.834*** [.752;.916]	.691*** [.583;.799]	.725*** [.622;.827]	.716*** [.612;.820]	1		
PPS	.451*** [.318;.584]	.268*** [.125;.412]	.331*** [.191;.472]	.304*** [.163;.446]	.360*** [.221;.499]	.268*** [.125;.412]	.449*** [.316;.582]	1	
DFS	.358*** [.219;.497]	.243** [.099;.387]	.294*** [.152;.437]	.354*** [.215;.494]	.385*** [.247;.522]	.397*** [.261;.534]	.462*** [.330;.594]	.446*** [.312;.579]	1

*Note.* ^t^ 0.05 < *p* < 0.1; * *p* < 0.05; ** *p* < 0.01; *** *p* < 0.001; UNC—Uncertainty; SFO—Self-Focus; BURD—Burden; CHA—Change; FIN—Finance; WOR—Worry; MPP—Total score for Motives for Postponing Parenthood: Total; PPS—Pure Procrastination Scale; DFS—Dark Future Scale.

### Discussion

As in the previous two studies, the main aim of Study 3 was to verify the internal structure of the English version of the MSMPP-18. The model evaluation displayed a good fit of the structural equation model to the data both in the 1^st^ order model and the 2^nd^ order model. Moreover, the two hypotheses (H9–H10) were entirely confirmed.

With respect to hypothesis H9, our research has clearly shown that postponing parenthood is positively correlated with procrastination.

This is consistent with what Steel and Ferrari [123] noted in a study of more than 20,000 people from English-speaking countries. Their results proved that people with severe procrastination (as measured by the *Irrational Procrastination Scale*; [121]) were less likely to form permanent partnerships and be married, and more likely to postpone parenthood or have fewer offspring than those who show less inclination to procrastinate. Taking a life-span motivational perspective and the fact that people in the current times are less determined by a normative calendar of life events, they may feel more pressure to make individual, meaningful, long-term life decisions. A wider time slot for the implementation of specific development tasks and less social expectation and pressure from the environment regarding the timing of developmental objectives may favor delaying their implementation. This, in turn, may activate procrastination tendencies as a self-regulation against failure [124]. This simultaneously explains why the hypothesis that postponed parenthood correlates negatively with self-regulation has been confirmed. Individuals who have greater self-regulation skills may also be less likely to postpone parenthood because their behavior is characterized by less procrastination through engaging in substitute activities. This may encourage researchers to examine in future studies a mediation model in which procrastination is the mechanism explaining the relationship between self-regulation and postponed parenthood.

Another circumstance that can promote procrastination is the potential longer life expectancy. This is highlighted by Kaftan and Freund [124], according to whom people nowadays on the one hand are given more social autonomy in some areas of life (such as marriage and family), and more social expectations in other domains (such as achieving education or building a career) on the other. This is why they may procrastinate on goals in which they are not judged and pressured by their environment. The authors aptly note, however, that associating postponing having a child with procrastination is only possible when the couple actually wants to have an offspring in the near future and is delaying conception despite having no contraindications to do so (e.g., financial), while being aware of the negative consequences of delay (e.g., declining fertility) that outweigh the subjective positive value of procrastination (e.g., achieving career goals).

Moreover, procrastination serves as a form of short-term mood enhancement and a maladaptive way of regulating emotions that causes a person to place a higher priority on improving the current mood than on achieving long-term behaviors and goals. The current mood then becomes more important than the consequences of not taking an action perceived as demanding (such as parenting) for the future self. Sometimes undertaking “structured procrastination” can be a way in which we “salvage” our self-image and our emotions by engaging in activities different from those intended; we achieve a sense of fulfillment and progress, improving well-being, but pushing back achieving our long-term commitments [125].

Hypothesis H10 was also confirmed. In fact, our study demonstrated a clear positive relationship between postponed parenthood and future anxiety. Previous research has already shown that women characterized by higher future anxiety have at the same time a more docile fear of childbirth [126]. Contemporary researchers addressing causes of deferring parenthood refer to a narrative framework that explains fertility postponement as a reaction to one’s own “narrative of the future” [127–129]. The significance, then, is not so much a person’s objective economic scenario, but their subjective perceptions of what the future will look like. A study by Gatta et al. [130] showed that men’s belief that they could regain their jobs after a possible loss was also important to their decision to become a parent. This shows that what matters is not only perceived job security, but perceived resilience to potential job loss. As Golovina and Jokela [131] show in their recent longitudinal study, an anxious perception of the future (e.g., in terms of economic instability, climate change, peace, immigration to Germany, hostility toward immigrants) is proven to be associated with both the likelihood of undertaking the role of a parent and the size of the family in a nationally representative German sample. This proves, as the authors of that study note, that not only the current situation of potential parents determines the decision to have children, but also their expectations and ideas about what the world will be like in the future. Among the potential explanations for this, they mention the influence of media reports about the war on the European continent and ongoing discussions about abstaining from offspring in the name of climate protection. Our research focused on an English-speaking sample from the United States, however, trends regarding climate change-motivated anti-natalism, among other things, have a global dimension [132–134].

The impact of fear of the future on parenting intentions can be selective, as shown in a study by Clark and Lepinteur [135]. Analyzing data from France, they showed that fear of future job security reduced the chance of having another offspring, but not parenthood in general. This shows the necessity of further research in this area, aimed at describing the mechanisms by which anxiety about the future is reflected in parenting decisions. This is especially the case since in some situations, uncertainty about the future paradoxically induces people to give birth to offspring, which is explained by a sense of influence over the immediate future (through uncertainty reduction, according to the uncertainty reduction approach [136]). The link between postponing parenthood and future anxiety may also stem from more general personality traits. In a study conducted by Pinquart et al. [137], individuals characterized by high levels of neuroticism presented greater ambivalence toward the prospect of parenthood. Similar results were obtained by Jokela et al. [138,139] on a sample of Finnish respondents.

## General discussion

The purpose of this research was to validate the original Polish version of the MSMPP-18 into English and ascertain whether three new datasets with English-speaking participants yield a goodness-of-fit indices. As far as we know, these three separate studies are the first attempt to validate the MSMPP-18 in a foreign language.

With regard to the six-dimensional structure of the MSMPP-18-EN (1: feeling of uncertainty and incompetence; 2: self-focus; 3: parenthood as a burden; 4: fear of change; 5: financial security concern; and 6: worry about a child’s future), the results confirmed its good characteristics. The CFA statistics provided empirical evidence that the multidimensional scale developed by Szcześniak et al. [38; Appendix] to measure motives of postponed parenthood has good fit indices across Studies 1–3, both for the first-order model and the second-order model. The six dimensions of the MSMPP-18-EN and its overall form displayed very good internal reliability across Studies 1–3.

Values of Cronbach’s alpha, McDonald’s Omega, and CR ranged: from 0.75 (fear of change) to 0.97 (financial security concern) in Study 1, from 0.68 (fear of change) to 0.93 (financial security concern and self-focus) in Study 2, and from 0.77 (fear of change) to 0.93 (financial security concern and self-focus) in Study 3. In fact, all estimates for research purposes exceeded the critical values of 0.70, with an exception in the case of the factor “fear of change” in Study 2. The lower reliability of the “fear of change” factor may be due to the fact that item 16 (“a woman’s body changes unfavorably after pregnancy”), which is part of it, has lower factor-loadings in the CFA analyses compared to results in the Polish version. Such a result may indicate that this item is more strongly influenced by culture and may result from variations in cultural ideals of beauty. Therefore, future research would be beneficial to examine the motives for deferred parenthood in the context of body image and self-objectification theory.

The second aim was to confirm the convergent validity of the MSMPP-18-EN. Since we had no other tools to measure the motives of deferred parenting, we used questionnaires that could share a common variance with the MSMPP-18-EN and align with it as similar constructs. Procrastination and future anxiety were found to be the most strongly and positively correlated variables with motives for delayed parenthood and its overall score (Study 3). Such a result is not surprising, because both the motives of postponed parenthood, and procrastination and future anxiety, are connected by uncertainty about what may happen in the future and, therefore, people may show difficulty in making decision to have a child. The other three constructs related positively to motives of postponed parenthood were: need for closure, negative emotions toward God, and family disfunction. This pattern of associations may suggest that people who tend to delay their decision to have a child strive for certainty, experience different forms of distress in the religious realm, or have deficits in their families. On the other hand, motives of postponed parenthood were negatively correlated with psychological capital, social support, positive emotions toward God, life satisfaction, self-efficacy, and self-regulation. Such a configuration of variables may imply that motives for postponing parenthood are associated with dimensions representing different areas of human functioning. It seems that such an important decision as starting a family relates to personal factors, which reflect people’s beliefs about their abilities to achieve goals, motivations in pursuing them, confidence about the future, and responsibility for their actions. The negative relationship between the motives for deferred parenthood and positive emotions toward God may suggest that experiencing one’s own religiosity and relationship with God is not indifferent to making important decisions about parenthood.

In the context of the nomological set obtained in these three studies, it can be assumed that the motives for deferred parenthood measured by the MSMPP-18-EN may be based, among others, on an intensive parenting ideology. If we consider that its essence lies in the tendency to feel anxiety about fulfilling parental roles toward the child as best as possible, the vision of oneself as a potential parent who must fulfill such expectations may lead to a sense of one’s own uncertainty or incompetence, fear of losing the opportunity for self-development, the feeling that parenthood is a burden, fear of change, financial concern, and worry about a child’s future. This is in line with Hays’ [140, p. x] description of this approach, which urges parents to “spend a tremendous amount of time, energy and money in raising their children.” If young people grow up with the “cultural script” [141] that parenting is about perfect devotion to a child and their needs, they may postpone having children until a time they consider more appropriate, i.e., when they have achieved greater maturity, self-fulfillment, and financial security.

An important aspect of our research concerns also the role that confounding variables may play in making decisions about parenthood. As the results show, these include age and intention to have a child in the future. It can be assumed that motives related to postponed parenthood are not only related to the variables verified in the hypotheses (family functioning, self-regulation, general self-efficacy, and satisfaction with life, emotions toward God, psychological capital, social support, need for closure, procrastination, and future anxiety), but also to age and the prospect of having children. With age and reflection on having children, the motives for postponing parenthood may lose their intensity.

Confirmation of the MSMPP-18’s structure in English corroborates, to a certain extent, its cross-cultural applicability. However, it should be critically noted that the replication of the structure in English may suggest a similarity of parenthood deferral motives in the broader Judeo-Christian Western culture, which shares similar values and ideals regarding parenting [142]. It is possible that in non-Western contexts, the structure of motives for postponement of the parenting role may be quite different [143,144]. A lack of cultural invariance has been shown, among others, for measurement of health [145], quality of life [146], anxiety and depression [147,148], and attachment [149]. This is all the more important because the measurement of parenting is particularly culturally entangled [150]. In such a situation, the applicability to Poland and English-speaking countries should be limited for the moment. However, further data are needed to demonstrate the universality of the scale in, for example, Spanish or German, as well as in more narrowly applied languages (such as Czech or Hungarian). At the same time, expanded research is required for other cultural circles (e.g., Far Eastern, Islamic), where the parenting model may be different [151,152].

Examining the motives for postponing parenthood using the MSMPP-18-EN can be helpful in a clinical context for individual and couples counseling. Just as the use of personality, depression and anxiety or work preference questionnaires allows for more relevant therapeutic interventions, similarly, discussing with a client or couple their individual motives for postponing parenthood can be helpful in better understanding themselves and their partner, and in working more constructively to overcome differences and potential difficulties [153]. In individual counseling, identifying the motives for postponing parenthood can help a person understand whether external (e.g., financial) or internal (e.g., anxiety) factors are more important in deciding whether to become a parent [154].

The application of the adapted scale in the context of social policy can help in two ways. On the one hand, from the perspective of departments responsible for family and social policy, it can be a useful tool for diagnosing the difficulties faced by those who defer parenthood, which can be addressed by those in power [155]. The motives for deferring the parental role, as this research shows, are varied and have either personal, developmental or social sources. Hence, the creation of more responsive policies can help overcome the difficulties of taking up parenthood for those who, due to external circumstances, decide to wait to have a child. On the other hand, studying the motives for postponing parenthood can be helpful in building social sensitivity toward those who feel cultural pressure to have a child, but have not made the final decision themselves. Building political and social conditions conducive to a thoughtful and voluntary decision can help these people create circumstances that are comfortable for a mature marriage/relationship and family planning [156].

### Limitations and future research

The presented validation of what is probably the first scale measuring the motives for deferred parenthood allows us to assume that the MSMPP-18 tool in the English version meets the theoretical and empirical criteria of a good questionnaire. However, the current three studies are not free from some limitations. First, although the MSMPP-18-EN has good psychometric properties confirmed by CFA indicators and correlations with constructs theoretically close to the measured motives, it does not exhaust the entire range of possible reasons why people postpone the decision to have a child. Future research could attempt to isolate other, equally important motives in the process of postponing parenthood (e.g., the experience of the family of origin, lack of a suitable partner, etc.). Second, any self-reported study has its limitations. In future research, quantitative analyses could also be accompanied by analyses based on qualitative material, to confirm the obtained results and enrich them with narrative elements. Third, the use of the CloudResearch platform is at times considered controversial in terms of representativeness and data quality control [157]. On the one hand, it provides precise group selection; however, it makes one question the data quality (e.g., the risk of using bots instead of humans; insufficient quality filters). However, research by Douglas et al. [158] indicates that the CloudResearch platform, among similar online data collection platforms, provides one of the highest rates of high-quality respondents and provides high-quality data. Nonetheless, future studies should take care to diversify the data collection methods used. Fourth, the selection of the variables for validating the tool was based on the availability of questionnaires measuring constructs that theoretically may seem close to the motives of deferred parenthood. Undoubtedly, the range of variables is much wider and in future research, it would be worth adding other personality, temperamental and social variables that would help to understand even more the motives for postponed parenthood. Another limitation concerns the inadequate control of the respondents’ level of religiosity in Study 2. Given that the *Emotions toward God Scale* was used, a certain shortcoming is the failure to relate the results to the respondents’ declared level of religiosity in that study. This is indicated by the differences in the mean and standard deviation between our study and the scale authors’ results [103]. In future studies, it would be a good idea to control for the subjective assessment of one’s own religiosity and other tools measuring this aspect of human functioning.

## Supporting information

S1 Table Supplementary material. (DOCX)
