# Supplementary material for: English validation of the Multidimensional Scale of Motives for Postponing Parenthood (MSMPP-18-EN): Factorial structure, psychometric properties, and correlates
Source: PLoS One. 2025 Aug 18;20(8):e0329404. doi: 10.1371/journal.pone.0329404 (PMC12360557; doi:10.1371/journal.pone.0329404)
Supplement: S1 Table — (DOCX) [file pone.0329404.s001.docx]

**Table 1: Supplementary material**

*Sociodemographic Characteristics of Participants at Study 1, 2 and 3*

|  | Study 1 | | Study 2 | | Study 3 | | Full sample | |
| --- | --- | --- | --- | --- | --- | --- | --- | --- |
|  | *n* | % | *n* | % | *n* | % | *n* | % |
| Gender | | | | | | | | |
| Female | 123 | 49.8 | 104 | 43.5 | 113 | 63.5 | 340 | 51.21 |
| Male | 97 | 39.3 | 125 | 52.3 | 63 | 35.4 | 285 | 42.92 |
| Agender | 1 | 0.4 | 1 | 0.4 | 0 | 0 | 2 | 0.30 |
| Non-binary | 11 | 4.4 | 7 | 2.9 | 0 | 0 | 18 | 2.71 |
| Other | 0 | 0 | 2 | 0.8 | 2 | 1.1 | 4 | 0.60 |
| Prefer not to say | 15 | 6.1 | 0 | 0 | 0 | 0 | 15 | 2.26 |
| Marital status | | | | | | | | |
| Married | 40 | 16.2 | 51 | 21.3 | n/a | n/a | n/a | n/a |
| Divorced | 10 | 4.0 | 3 | 1.3 | n/a | n/a | n/a | n/a |
| Separated | 2 | 0.8 | 2 | 0.8 | n/a | n/a | n/a | n/a |
| In a civil union/partnership | 3 | 1.2 | 4 | 1.7 | n/a | n/a | n/a | n/a |
| In a relationship | 72 | 29.1 | 57 | 23.8 | n/a | n/a | n/a | n/a |
| Single | 107 | 43.3 | 122 | 51.0 | n/a | n/a | n/a | n/a |
| Prefer not to say | 13 | 5.3 | 0 | 0 | n/a | n/a | n/a | n/a |
| Ethnicity | | | | | | | | |
| White/Caucasian | 172 | 69.6 | 156 | 65.3 | 115 | 64.6 | 66.72 | 66.72 |
| Black/African American | 30 | 12.1 | 31 | 13.0 | 6 | 3.4 | 10.09 | 10.09 |
| American Indian/Native American | 4 | 1.6 | 2 | 0.8 | 10 | 5.6 | 2.41 | 2.41 |
| Hispanic | 16 | 6.5 | 19 | 7.9 | 21 | 11.8 | 8.43 | 8.43 |
| Asian/Pacific Islander | 17 | 6.9 | 23 | 9.6 | 16 | 9.0 | 8.43 | 8.43 |
| Middle Eastern | 1 | 0.4 | 1 | 0.4 | 4 | 2.2 | 0.90 | 0090 |
| Other | 7 | 2.8 | 7 | 2.9 | 6 | 3.4 | 3.01 | 3.01 |
| Highest educational level | | | | | | | | |
| A 4-year degree | 73 | 29.6 | 94 | 39.3 | 48 | 27.0 | 215 | 32.38 |
| A 2-year degree | 31 | 12.6 | 33 | 13.8 | 6 | 3.4 | 70 | 10.54 |
| A master's degree or higher | 26 | 10.5 | 36 | 15.1 | 42 | 23.6 | 104 | 15.66 |
| College or trade school | 33 | 13.4 | 17 | 7.1 | 24 | 13.5 | 74 | 11.14 |
| High school | 84 | 34.0 | 59 | 24.7 | 58 | 32.6 | 201 | 30.27 |
| What is the most appropriate age to have your first child? | | | | | | | | |
| 18-20 | 3 | 0 | 3 | 1.3 | 0 | 0 | 6 | 0.90 |
| 21-23 | 5 | 2.0 | 4 | 1.7 | 2 | 1.1 | 11 | 1.66 |
| 24-26 | 45 | 18.2 | 31 | 13.0 | 24 | 13.5 | 100 | 15.06 |
| 27-29 | 76 | 30.8 | 71 | 29.7 | 66 | 37.1 | 213 | 32.08 |
| 30-32 | 65 | 26.3 | 72 | 30.1 | 49 | 27.5 | 186 | 28.01 |
| 33-35 | 26 | 10.5 | 35 | 14.6 | 22 | 12.4 | 83 | 12.50 |
| 36-38 | 13 | 5.3 | 10 | 4.2 | 3 | 1.7 | 26 | 3.92 |
| 39-41 | 4 | 1.6 | 5 | 2.1 | 1 | 0.6 | 10 | 1.51 |
| More than 41 | 2 | 0.8 | 2 | 0.8 | 2 | 1.1 | 6 | 0.90 |
| Prefer not to say | 8 | 3.2 | 6 | 2.5 | 9 | 5.1 | 23 | 3.46 |
| Would you like to have children in the future? | | | | | | | | |
| Yes | 147 | 59.5 | 161 | 67.4 | 132 | 74.2 | 440 | 66.27 |
| No | 100 | 40.5 | 78 | 32.6 | 46 | 25.8 | 224 | 33.73 |
| Number of children you would like to have | | | | | | | | |
| 1 child | 48 | 19.4 | 58 | 24.3 | 15 | 8.5 | 121 | 18.22 |
| 2 children | 80 | 32.4 | 79 | 33.1 | 73 | 41 | 232 | 34.94 |
| 3 children | 16 | 6.5 | 27 | 11.3 | 33 | 18.6 | 76 | 11.45 |
| 4 or more | 7 | 2.8 | 5 | 2.1 | 13 | 7.4 | 25 | 3.77 |
| No response | 96 | 38.9 | 70 | 29.3 | 44 | 24.5 | 210 | 31.63 |

*Note. Study 1: N* = 247; Mean age was 32.24 years old (*SD* = 7.14); Study 2: *N* = 239; Mean age was 31.92 years old (*SD* = 6.82); Study 3: *N* = 178; Mean age was 25.42 years old (*SD* = 5.80).
